# European Headache Federation (EHF) critical re-appraisal and meta-analysis of oral drugs in migraine prevention - part 4: propranolol

**DOI:** 10.1186/s10194-024-01826-y

**Published:** 2024-07-24

**Authors:** Jan Versijpt, Christina Deligianni, Muizz Hussain, Faisal Amin, Uwe Reuter, Margarita Sanchez-del-Rio, Derya Uluduz, Deirdre Boucherie, Dena Zeraatkar, Antoinette MaassenVanDenBrink, Simona Sacco, Christian Lampl, Raquel Gil-Gouveia

**Affiliations:** 1 Department of Neurology, Vrije Universiteit Brussel (VUB), Universitair Ziekenhuis Brussel (UZ Brussel), Brussels, Belgium; 2 Department of Neurology, Athens Naval Hospital, Athens, Greece; 3 Department of Anesthesia and Department of Health Research Methods, Evidence and Impact, McMaster University, Hamilton, Canada; 4 Department of Neurology, Danish Headache Center, Copenhagen University Hospital – Rigshospitalet, Copenhagen, Denmark; 5 Department of Neurology, Charité Universitätsmedizin Berlin, Berlin, Germany; 6 Department of Neurology, Clinica Universidad de Navarra, Madrid, Spain; 7 Department of Neurology, Istanbul University Istanbul Faculty of Medicine, Istanbul, Turkey; 8 Department of Internal Medicine, Division of Vascular Medicine and Pharmacology, Erasmus MC Medical Center, Rotterdam, the Netherlands; 9 Department of Biotechnological and Applied Clinical Sciences, University of L´Aquila, Rome, Italy; 10 Department of Neurology and Stroke Unit Konventhospital Barmherzige Brüder Linz, Linz, Austria; 11 Neurology Department, Hospital da Luz Headache Center, Hospital da Luz Lisboa, Lisbon, Portugal

## Abstract

**Objective:**

The aim of this paper is to critically re-appraise the published trials assessing propranolol for migraine prophylaxis.

**Methods:**

We report methods and results following the Preferred Reporting Items for Systematic Reviews (PRISMA), by searching MEDLINE, EMBASE, Cochrane CENTRAL, and ClinicalTrials.gov for randomized trials of pharmacologic treatments for migraine prophylaxis. We included randomized trials that compared propranolol with placebo for migraine prophylaxis in adults. The outcomes of interest were informed by the Core outcome set for preventive intervention trials in chronic and episodic migraine (COSMIG) and include the proportion of patients who experience a 50% or more reduction in monthly migraine days, the reduction of monthly migraine days, and the number of adverse events leading to discontinuation. We assessed risk of bias by using a modified Cochrane RoB (risk of bias) 2.0 tool and the certainty of evidence by using the GRADE approach.

**Results:**

Our search yielded twenty trials (*n* = 1291 patients) eligible for data synthesis and analysis. The analysis revealed a moderate certainty evidence that propranolol leads to a reduction in monthly migraine days versus placebo (-1.27; 95% CI: -2.25 to -0.3). We found moderate certainty evidence that propranolol increases the proportion of patients who experience a 50% or more reduction in monthly migraine days, compared to placebo with a relative risk of 1.65 (95% CI 1.41 to 1.93); absolute risk difference: 179 more per 1,000 (95% CI 113 to 256). We found high certainty evidence that propranolol increases the proportion of patients who discontinue due to adverse events compared to placebo with a risk difference of 0.02 (95% CI 0.00 to 0.03); absolute risk difference: 20 more per 1,000 (95% CI 0 to 30).

**Conclusions:**

The present meta-analysis shows that propranolol has a prophylactic role in migraine, with an overall acceptable tolerability profile. Combining these results with its long-standing use and its global availability at a low cost confirms its role as a first line agent in the prophylaxis of migraine.

## Introduction

Beta-blockers have a well-established history as prophylactic treatments for migraine, dating back to the late 1960s when propranolol was incidentally discovered to be effective in migraine prevention [1]. This discovery led to several clinical trials on the use of beta-blockers for migraine prophylaxis in the 1970s [2]. Beta-blockers have ever since become widely used treatment options and are still recommended as first-line treatments for migraine prophylaxis in all major treatment guidelines due to their established efficacy, safety profile, widespread availability and affordability [3–5].

Beta-blockers are antagonists of the β_1(/2)_-adrenoceptors, which are G-coupled protein receptors activated by catecholamines such as (nor)adrenaline. The most conspicuous mechanism of action is on the cardiovascular system, where blockade of β-adrenergic receptors leads via antagonism of the β_1_-adrenoceptor to a decrease in sympathetic activity, resulting in a decrease in heart rate and subsequent decrease in blood pressure [6, 7]. In addition, beta-blockers that also display affinity for the β_2_-adrenoceptor may induce a decrease in peripheral vasodilation [8].

**Fig. 1:**
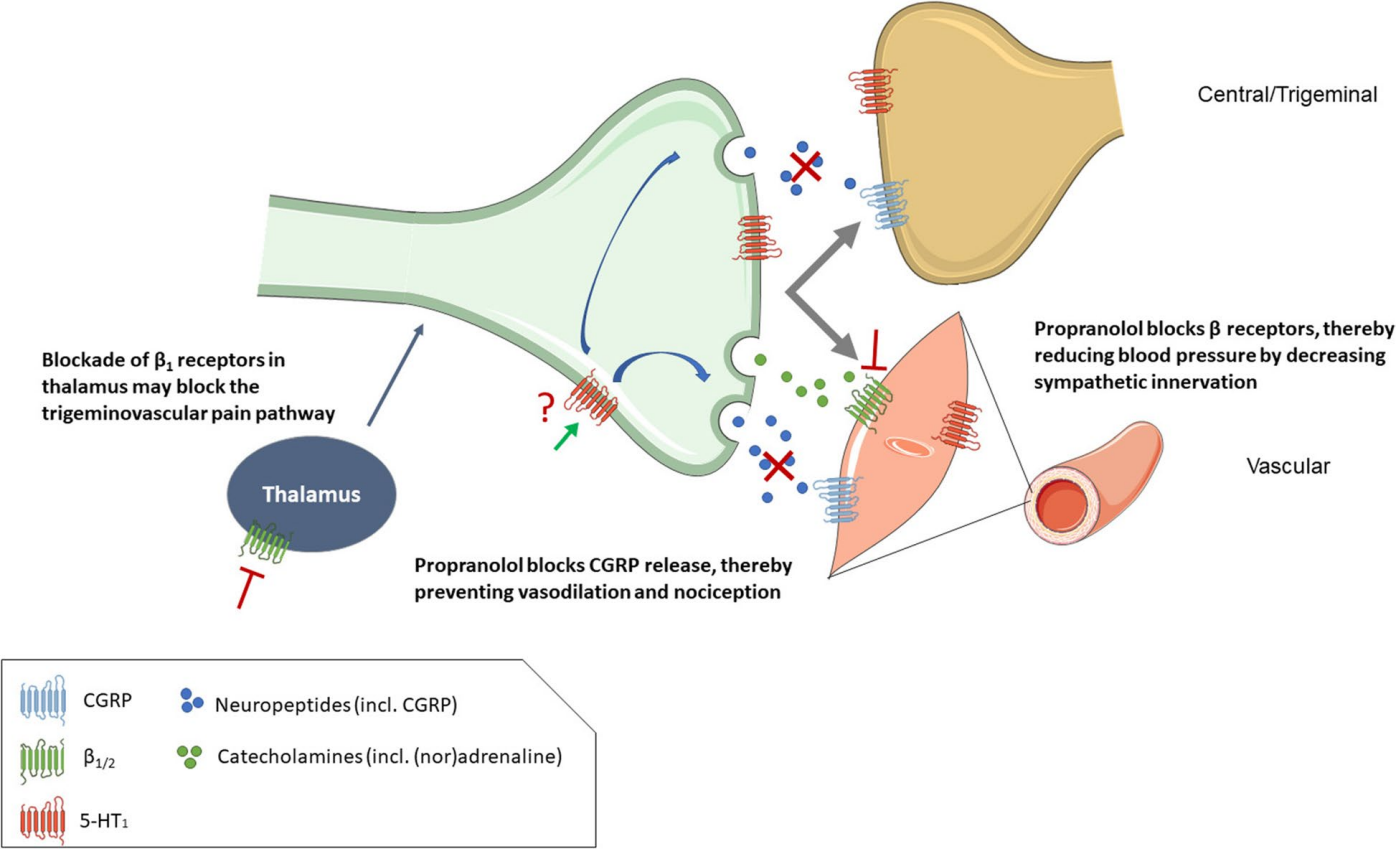
Potential mechanisms of action for the anti-migraine effect of the β_1/2_ receptor antagonist propranolol. By blocking β-adrenergic receptors, propranolol reduces blood pressure by decreasing sympathetic innervation [6, 7]. Furthermore, blockade of β_1_-adrenergic receptors in the thalamus may block the trigeminovascular pain pathway, however, there are contradicting findings on the importance of this potential pathway. Propranolol can block capsaicin-mediated vasodilation mediated by the trigeminal nerve, possibly through agonism of presynaptic 5-HT_1_ receptors [9]. Activation of 5-HT_1_ receptors can block release of CGRP, leading to reduced vasodilation and nociception

Although the precise underlying mechanisms of the antimigraine effect of beta-blockers remain uncertain, several potential mechanisms of action have been proposed. One mechanism is the inhibition of the trigeminovascular system, as β1-adrenoceptor antagonism blocks trigeminovascular nociception in the ventroposteromedial nucleus of the thalamus. Moreover, propranolol can also block capsaicin-induced increases in trigeminally-innervated dermal blood flow unrelated to cardiovascular effects, possibly through agonism of presynaptic 5-HT_1_ receptors [9, 10]. Another potential mechanism involves the suppression of cortical spreading depression (CSD), as observed in a rat model [11], which may relate to altered neurotransmission in migraine pathways in the brain, thereby raising the attack threshold (Fig. 1).

Although beta-blockers are thought to be effective in migraine prophylaxis as a class, a meta-analysis has demonstrated that propranolol is the most efficacious, while metoprolol is likely effective. In contrast, atenolol, bisoprolol, and timolol had only weak evidence of benefit, and acebutolol, alprenolol, and nadolol were found to be ineffective [12]. Overall however, due to limited trial data for most beta-blockers particularly those studied in fewer than three trials, the conclusions regarding their efficacy are less certain [12]. The variability in effectiveness may also be attributed to differences in selectivity or competitiveness for β_1/2_-adrenoceptors, lipid solubility and ability to cross the BBB, affinities for 5-hydroxytryptamine (serotonin or 5-HT) 5-HT_1_, 5-HT_2B_ and 5-HT_2C_ r$eceptors, and/or propranolol’s ability to block inducible nitric oxide synthase (iNOS) [13]. Most interestingly, some beta-blockers, including propranolol, have also been described to act as agonists at the 5-HT_1B/1D_ receptor, and might thus have a presynaptic inhibiting effect on CGRP release, similar as the triptans have [14].

The aim of this study was to conduct a systematic review and a meta-analysis of published trials to evaluate the efficacy of propranolol compared to placebo in the prophylactic management of migraine. This re-appraisal is part of a larger work initiated by the EHF where the same effort has already been done for flunarizine, amitriptyline and topiramate [15–17].

## Methods

This work is the fourth study of the EHF series aiming to re-appraise different types of classic migraine preventive medications, so the methods for this review have been previously described in detail [15–17].

In summary, we report our methods and results following the Preferred Reporting Items for Systematic Reviews (PRISMA) [18].

### Search strategy

In consultation with an experienced research librarian, we searched MEDLINE, EMBASE, Cochrane CENTRAL, and ClinicalTrials.gov from inception to August 13, 2022 for randomized trials of pharmacologic treatments for migraine prophylaxis, without language restrictions. We supplemented our search by retrieving references of similar systematic reviews and meta-analyses [12].

### Screening and study eligibility

Following training and calibration exercises to ensure sufficient agreement, pairs of reviewers, working independently and in duplicate, reviewed titles and abstracts of search records and subsequently the full-text of records deemed potentially eligible at the title and abstract screening stage. Reviewers resolved discrepancies by discussion, or, when necessary, by adjudication with a third viewer. We included randomized trials that compared propranolol with placebo for migraine prophylaxis in adults. We excluded trials that investigated abortive rather than prophylactic interventions and trials that randomized children or adolescents. We excluded trials that randomized fewer than 25 participants. We anticipated that smaller trials may be unrepresentative and at higher risk of publication bias [19].

### Data extraction

Following training and calibration exercises to ensure sufficient agreement, pairs of reviewers worked independently and in duplicate to collect data from eligible trials using a pilot-tested Excel spreadsheet (Microsoft Office Excel 2019). Reviewers resolved discrepancies by discussion and if necessary, by adjudication with a third party. We extracted trial characteristics, patient characteristics, diagnostic criteria, type of migraine, intervention characteristics, and outcomes of interest at the longest reported follow-up time at which patients were still using the interventions being investigated. Our outcomes of interest were informed by the Core outcome set for preventive intervention trials in chronic and episodic migraine (COSMIG) and include the reduction of migraine days per month, the proportion of patients who experience a 50% or more reduction in migraine days per month, and the number of adverse events leading to discontinuation [20]. We prioritized extracting monthly migraine days when reported but also extracted monthly headache days or monthly migraine attacks when monthly migraine days were not reported. We acknowledge that there may be heterogeneity in how trials define migraine and headache days. For example, monthly migraine days may be averaged over a single month or extrapolated based on measurements of migraine days over several weeks. In such cases, we pooled results despite heterogeneity in outcome definitions. We anticipated that since both the intervention and comparator arm outcome would be measured similarly, the mean difference between them - the statistic meta-analyzed - would be consistent across trials. For all outcomes, we extracted results at the longest reported point of follow-up. We identified one crossover trial, which we treated as a parallel group trial in meta-analyses, an overall conservative approach to including data from crossover trials since it does not account for correlation within individuals and reduces the weight of the trial in the meta-analysis.

### Risk of bias assessments

Following training and calibration to ensure sufficient agreement, reviewers working independently and in duplicate, assessed the risk of bias using a modified Cochrane RoB 2.0 tool [21]. For each trial, we rated each outcome as either ‘low risk of bias’, ‘some concerns–probably low risk of bias’, ‘some concerns–probably high risk of bias’, and ‘high risk of bias’ across the following domains: bias arising from the randomization process, bias due to departures from the intended intervention, bias due to missing outcome data, bias in the measurement of the outcome, and bias in the selection of the reported results. Reviewers resolved discrepancies by discussion and if necessary, by adjudication with a third reviewer.

### Data synthesis and analysis

For all outcomes, we performed frequentist random-effects meta-analysis using the restricted maximum likelihood (REML) estimator. We analyzed a 50% or more reduction in monthly migraine days as relative risks, the reduction in monthly migraine days as mean differences, and the number of adverse events leading to discontinuation as risk differences. To facilitate interpretation, we report dichotomous outcomes as number of events per 1,000 patients.

We summarize heterogeneity using the I^2^ statistic and interpret an I^2^ value of 0% to 40% as not important, 30% to 60% as moderate heterogeneity, 50% to 90% as substantial heterogeneity, and 75% to 100% as considerable heterogeneity [22].

We anticipated that the effects of treatments may vary based on the risk of bias, baseline monthly migraine days, and the proportion of patients that had previously used prophylactic therapy. To test for subgroup effects based on these factors, we performed pairwise meta-regressions comparing results rated at low versus high risk of bias and trials below versus above the median number of monthly migraine days or proportion of patients that had previously used prophylactic therapy.

Inferences of effect modification, also known as subgroup effects, often prove spurious. Such spurious claims may be attributed to testing many factors, leading to apparent but inaccurate evidence of effect modification due to chance, selective reporting, or improper statistical analysis. To avoid misleading claims of effect modification, we assessed the credibility of subgroup effects using the ICEMAN tool - the gold standard tool for evaluating effect modification [23].

We performed all analyses using the *meta* and *metafor* packages in R (version 4.1.2) [24].

### Assessment of the certainty (quality) of evidence

We assessed the certainty of evidence using the GRADE approach [25]. For each outcome, we rated the certainty of each comparison as either high, moderate, low, or very low based on the risk of bias, inconsistency, indirectness, imprecision, and publication bias. We made judgements of imprecision using the minimally contextualized approach [26]. The minimally contextualised approach considers only whether confidence intervals include the null effect and thus does not consider whether plausible effects, captured by confidence intervals, include both important and trivial effects. To evaluate the certainty of no effect, we used minimally important differences, sourced from the literature and by consensus from the authors. We considered a 15% increase in the proportion of patients who experienced a 50% or more reduction in monthly migraine days, 2 monthly migraine days, and a 2% increase in patients who experienced adverse events leading to discontinuation as minimally important.

We report results using GRADE simple language summaries (i.e., describing high certainty evidence with declarative statements, moderate certainty evidence with ‘probably’, low certainty evidence with ‘may’ and very low indicated by ‘very uncertain’) [27].

## Results

### Data synthesis and analysis

**Fig. 2:**
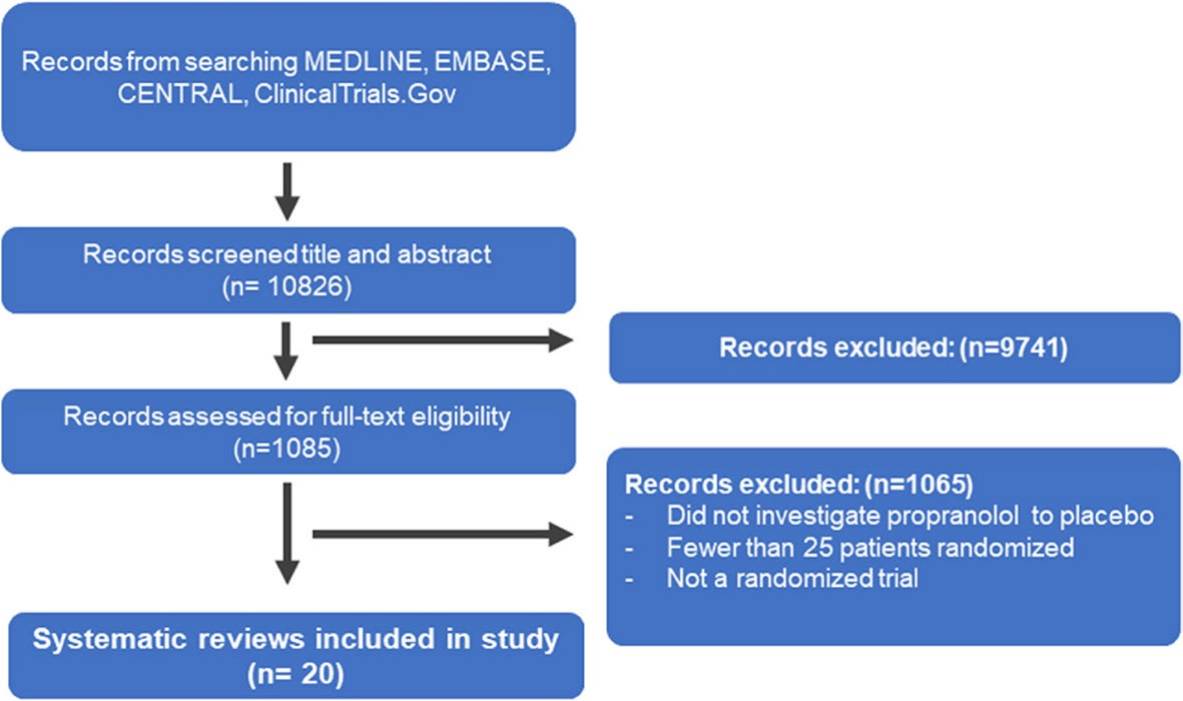
Selection of studies for the systematic review

Our search yielded 10,826 records. Fifty-nine trials were identified that investigated propranolol for migraine. Thirty-seven were excluded because they did not include a placebo arm or randomized fewer than 25 patients and two were excluded because they were not randomized. Twenty trials met the inclusion criteria and were used for quantitative analysis, covering a period from 1972 to 2014 [2, 28–45]. Figure 2 presents details about study selection. Among the trials that were included, 10 of them used the definition of migraine from the Ad hoc committee on the classification of headache [46], while only 6 used the International Classification of Headache Disorders [47]. The remaining trials did not provide information on migraine diagnostic criteria. Most trials were single center studies (*n* = 13, 65%) and were conducted in the USA (6), Scandinavia (5), the UK (1) and New Zealand (1). Out of the seven multicenter trials, only two were international, while the other multicenter trials were conducted in Scandinavia (2), the USA (2), and France (1).

**Fig. 3:**
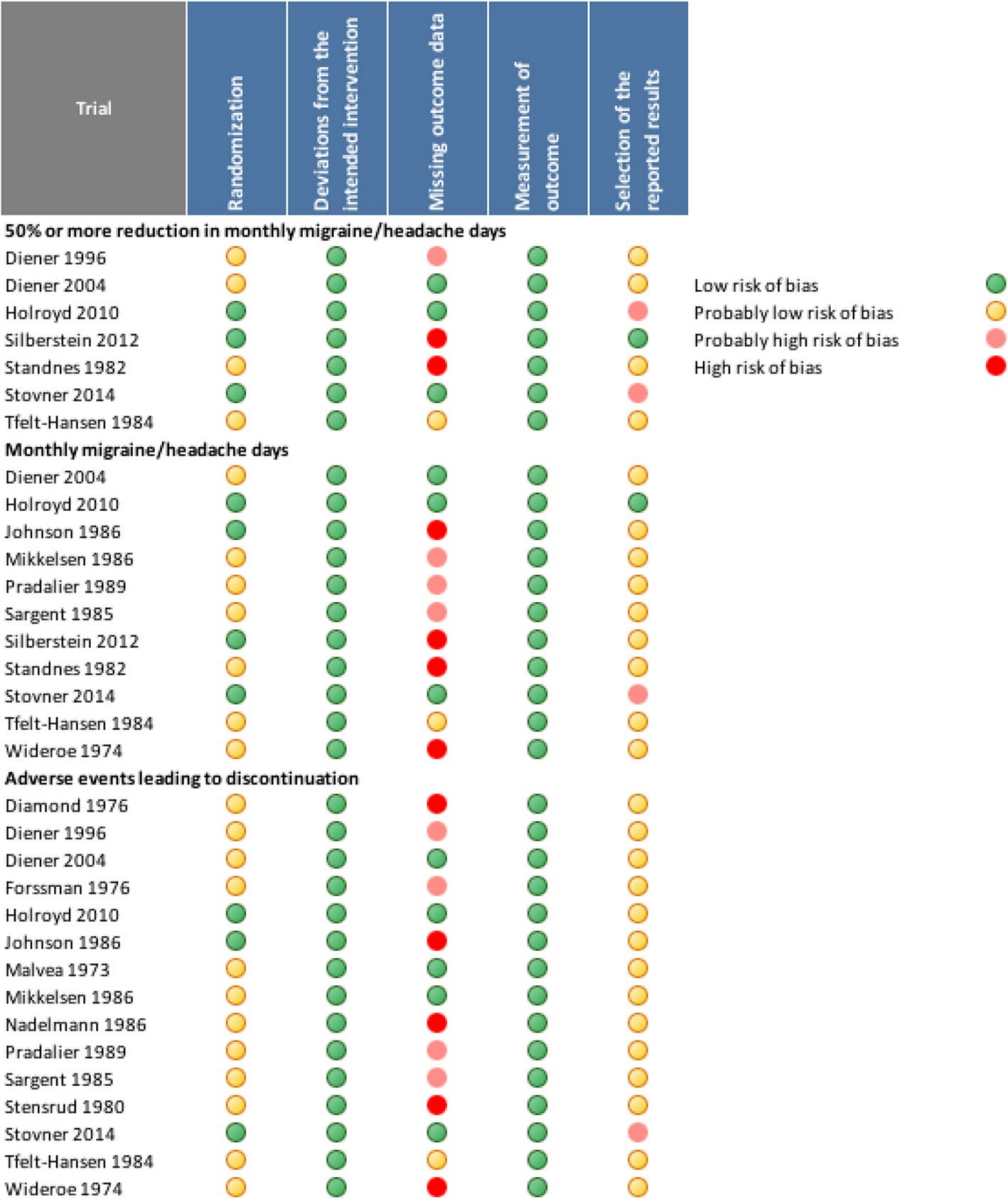
Risk of bias assessment

In total, 1291 participants, with an average age of 38.6 years, were included in these studies, with females comprising 78.9% of the sample. Propranolol daily dosages varied between 60 and 320 mg and the study duration varied between 4 and 24 months. The risk of bias of the eligible trials is presented in Fig. 3.

### Monthly migraine days

**Fig. 4:**
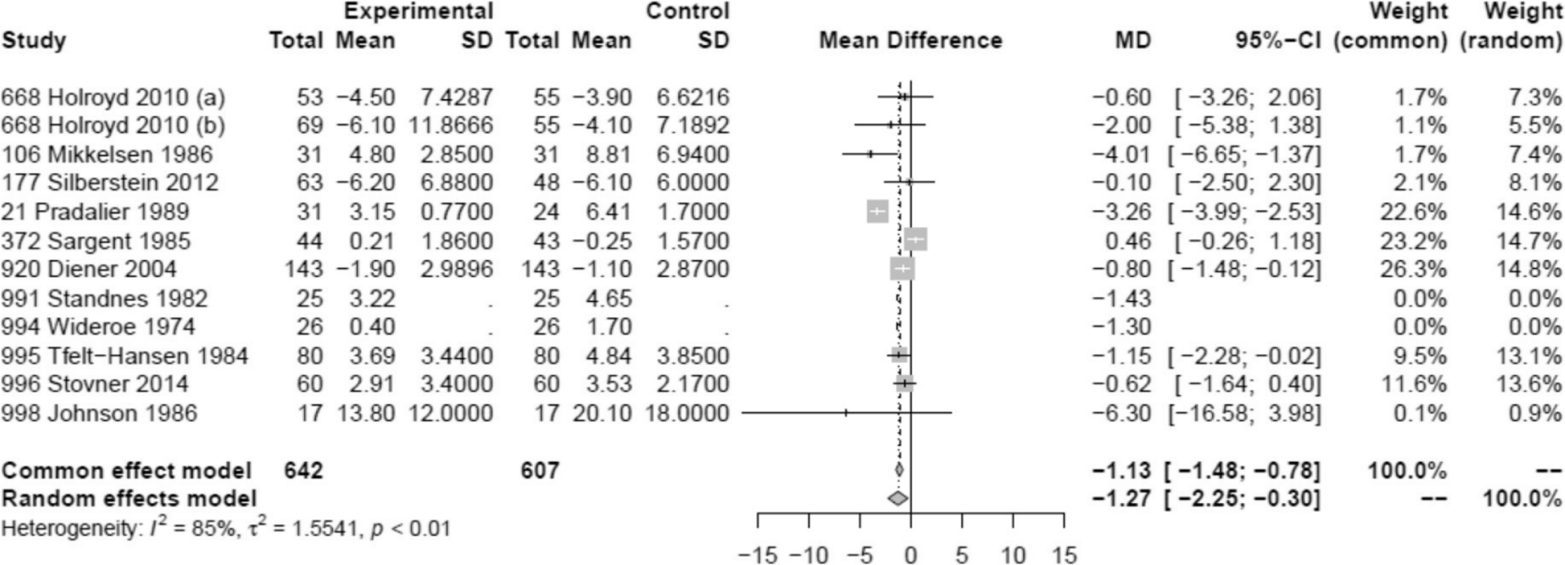
Forest plot of analysis comparing propranolol with placebo for the reduction in monthly migraine days

Twelve trials, including 642 patients, reported on the reduction in monthly migraine days [2, 31, 33, 35, 37–40, 42, 43, 48]. We found moderate certainty evidence that propranolol reduces monthly migraine days with a mean difference of 1.27 days (95% CI 0.3 to 2.25) compared to placebo. Only three of these trials were considered as (probably) low risk of bias [31, 33, 43] (Figs. 3 and 4, Table 1).

**Table 1:** Propranolol compared to placebo for migraine prophylaxis

**Propranolol compared to placebo for migraine prophylaxis**					
**Patient or population: **migraine **Intervention: **prophylaxis with propranolol **Comparison: **placebo					
**Outcomes**	**№ of participants (studies)** **Follow-up**	**Certainty of the evidence** **(GRADE)**	**Relative effect** **(95% CI)**	**Anticipated absolute effects**	
				**Risk with placebo**	**Risk difference with propranolol**
50% or more reduction in monthly migraine days	982 (7 RCTs)	**Moderate** (downgraded due to risk of bias)	**RR 1.65** (1.41 to 1.93)	275 per 1,000	**179 more per 1,000** (113 more to 256 more)
Monthly migraine days	935 (10 RCTs)	**Moderate** (downgraded due to risk of bias)	-	NA	**MD** **1.27 migraine days fewer** (2.25 fewer to 0.3 fewer)
Adverse events leading to discontinuation	1,291 (15 RCTs)	**High**	**RD 0.02** (0.00 to 0.03)	0 per 1,000	**20 more per 1,000** (0 more to 30 more)

*CI* confidence interval, *MD* mean difference, *RR* risk ratio, *RD* risk difference

GRADE Working Group grades of evidence

*High certainty*: we are very confident that the true effect lies close to that of the estimate of the effect

*Moderate certainty*: we are moderately confident in the effect estimate: the true effect is likely to be close to the estimate of the effect, but there is a possibility that it is substantially different

*Low certainty*: our confidence in the effect estimate is limited: the true effect may be substantially different from the estimate of the effect

*Very low certainty*: we have very little confidence in the effect estimate: the true effect is likely to be substantially different from the estimate of effect

### 50% responder rate

**Fig. 5:**
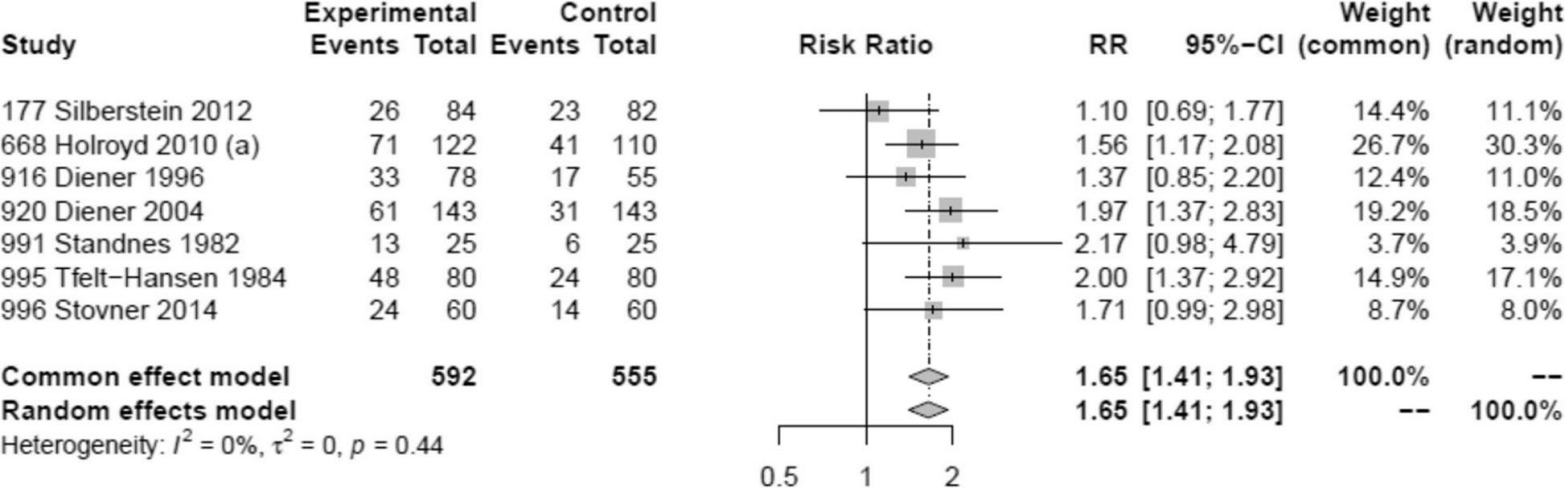
Forest plot of analysis comparing propranolol with placebo for 50% responder rate

Seven trials reported on 50% or more reduction in monthly migraine days comprising a total of 592 patients [30, 31, 33, 39, 40, 42, 43]. Overall, we found moderate certainty evidence that propranolol probably increases the proportion of patients who experience a 50% or more reduction in monthly migraine days, compared to placebo (Fig. 5). The certainty of evidence was downgraded by one level due to concerns about the risk of bias (Fig. 3). Across eligible trials, the relative effect of propranolol compared to placebo was 1.65 (95% CI 1.41 to 1.93) (Table 1).

### Adverse events leading to discontinuation

**Fig. 6:**
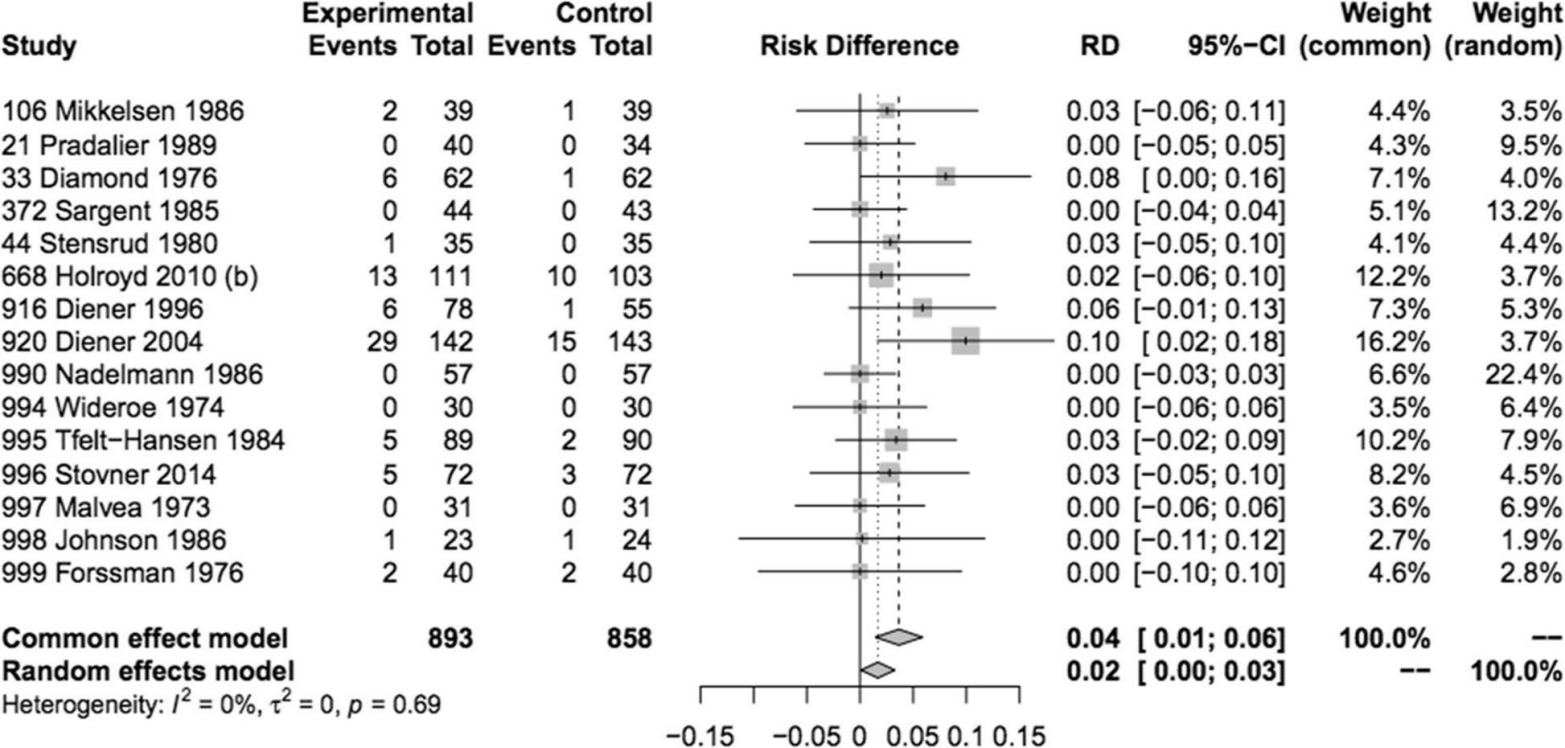
Forest plot of analysis comparing propranolol with placebo for adverse events leading to discontinuation

Adverse events leading to discontinuation could be assessed in 15 trials involving 893 subjects, with a higher incidence observed in those receiving propranolol compared to those receiving placebo, with a risk difference of 20 per 1000 (Fig. 6, Table 1). Only 5 trials were considered as (probably) low risk of bias (Fig. 3). Nevertheless, this lesser tolerance of propranolol received a high level of certainty GRADE rating. In the propranolol group, 70 patients (8%, versus 4% with placebo) discontinued treatment due to side effects. Out of these, four were considered serious adverse events (SAEs), being one case of hepatitis and three cases of incident pregnancies. The most commonly reported adverse events were tiredness/reduced physical capacity (32.1% of reported AEs), nausea/vomiting (11.4%), insomnia/sleep disturbances including nightmares or vivid dreams (9.1%), dizziness (6.7%), bodily, neck, or low back pain (6.5%), and numbness (5.7%). Other adverse events reported included symptoms related to the gastric tract (heartburn or unspecified pain, diarrhea/constipation), cardiovascular symptoms (low heart rate, low blood pressure or palpitations), depression, somnolence, malaise, appetite changes (increased/decreased), weight changes (gain/loss), cognitive symptoms related to attention and memory and to a lesser extent Raynaud phenomenon and bronchospasm.

## Discussion

The results of this meta-analysis confirm the efficacy of propranolol for the prevention of migraine based on both monthly migraine days and 50% responder rate, both outcomes graded with a moderate certainty GRADE evidence. Comparing the results to a previously performed meta-analysis performed by Jackson et al. we can conclude that the magnitude of effect is in line with the present findings [12]. Small difference could be related to the fact that the former meta-analysis looked at beta-blockers as a class and, moreover, a slightly different endpoint was used (headaches/month). Putting results in a larger perspective, both the magnitude of clinical efficacy and its adverse events leading to discontinuation of propranolol are in line with results found with current CGRP-based preventive treatments [49]. The latter however are only available at a significantly higher cost or, worse, are not even affordable by a public health system. Based on the above, the prominent place of propranolol in most guidelines where it is generally considered as one of or even the first line prophylactic agent in migraine seems justified. The latter is reinforced by the fact that propranolol was the first agent providing evidence for prophylaxis in migraine, its ease of use, global availability and its low cost. This ease of use and global availability at a low cost is critical given the enormous challenges that lie ahead of us related to the global headache burden [50]. In light of this global burden, one should indeed be aware of the fact that the large majority not receiving preventive therapy for migraine originate from low-to-medium-income countries [51, 52].

With 15 randomized controlled trials providing efficacy data for propranolol, this agent largely outnumbers all other prophylactic agents in migraine concerning the number of trials. However, one needs to be aware of the fact that most of these trials are small, prone to several risks of bias and were not performed in line with recent guidelines on drug trials in migraine prevention [53]. Also, most if not all evidence in migraine prophylaxis is gathered in episodic migraine. Only one recent study evaluated the efficacy of propranolol versus topiramate in chronic migraine where both yielded similar efficacy results [54]. This lack of evidence in chronic migraine is true for almost all conventional prophylactic drugs except partly for topiramate. The primary reason for this is the relatively recent recognition of chronic migraine as a distinct condition, with its current diagnostic criteria incorporated into the International Classification of Headache Disorders (ICHD) as recently as 2013 [55].

The effectiveness of propranolol in migraine is further substantiated by efficacy data of several other beta-blocking agents either based on randomized placebo-controlled trials or comparative studies. Indeed, also metoprolol and to a lesser extent atenolol, bisoprolol, timolol, nadolol, nebivolol and pindolol are therefore often used in the prophylaxis of migraine. In fact, it is generally considered that all beta-blockers have prophylactic potential in migraine except most probably those with intrinsic sympathetic activity like acebutolol. However, this assumption is only partly substantiated by evidence based on randomized controlled clinical trials [56, 57].

The present meta-analysis concluded with high certainty that propranolol leads to more discontinuations due to side-effects compared to placebo. The magnitude of this effect seems small however, certainly when compared to other oral drugs used in migraine prevention like topiramate, amitriptyline and valproate [58, 59]. Moreover, no SAEs were reported in any of the eligible trials. All this reflects clinical practice where propranolol is overall well tolerated on average and serious side-effects are almost non-existent. The different characteristics of other beta-blockers can even be used in case of side-effects without losing efficacy e.g. switching to a cardioselective beta-blocker like metoprolol in case of (a history of) asthmatic disease and to a lesser extent obstructive pulmonary disease [60]. Similarly, atenolol can be used in case of sleep disturbances since its lower lipophilicity results in limited BBB penetrance, although other mechanisms might be involved in central nervous system side effects as well [61]. Finally, nebivolol is unlikely to cause Raynaud’s phenomenon due to its vasodilating capacity [62].

In conclusion, the present meta-analysis confirms that propranolol has a prophylactic role in migraine, although the trials providing evidence for this vary widely in terms of quality and are often prone to bias. Furthermore, propranolol demonstrates a favorable safety profile. Considering also its extensive history of use and widespread availability at a low cost, its placement as a first-line therapeutic agent in migraine prophylaxis seems justified.

## Data Availability

No datasets were generated or analysed during the current study.

## Abbreviations

AEs: Adverse events
BBB: Blood brain barrier
Ca^2+^: Calcium cations
CGRP: Calcitonin gene-related peptide
FDA: Food and Drug Administration
CSD: Cortical spreading depression
ICHD: International Classification of Headache Disorders
PRISMA: Preferred Reporting Items for Systematic Reviews
COSMIG: Outcome Set for preventive intervention trials in chronic and episodic migraine
RCT: Randomised Controlled Trial
SAE: Serious adverse event
